# Editorial: Role of Macrophage MicroRNAs in Inflammatory Diseases and Cancer

**DOI:** 10.3389/fimmu.2021.764525

**Published:** 2021-09-13

**Authors:** Yves Renaudineau, Ioana Berindan-Neagoe, Luminita Aurelia Stanciu

**Affiliations:** ^1^Laboratory of Immunology, CHU TOULOUSE, INSERM UMR1291 - CNRS UMR5051 - Université Toulouse III, Toulouse, France; ^2^Research Center for Functional Genomics, Biomedicine and Translational Medicine, Iuliu Hatieganu University of Medicine and Pharmacy, Cluj-Napoca, Romania; ^3^National Heart & Lung Institute, Imperial College London, London, United Kingdom

**Keywords:** macrophages, microRNAs, cancer, inflammatory diseases, M1- or M2-macrophages

Macrophages are important players in immune pathogenesis and the miRNA macrophage involvement was proved to be central in various human diseases from hematologic malignancies and disorders, solid tumors, to allergy, asthma and autoimmune diseases. Indeed, MicroRNAs (miRNA) are short non-coding RNAs involved in regulating the differentiation between the pro-inflammatory type 1 macrophages (M1) and the anti-inflammatory type 2 macrophages (M2). Such effect can be direct (endogenous miRNA) or indirect when exogenous miRNA (including ones produce by macrophages) can influence the balance between type 1 and type 2 cytokines in their tissue environment (1). To this end it has been demonstrated that exosomal miRNA could be transferred from macrophages to other cell types, and vice versa, *via* extracellular vesicles influencing their phenotype and functions.

This Research Topic papers (original research paper and reviews) provided knowledge on the role of miRNA macrophage in inflammatory diseases and cancer where monocyte/macrophage play an important role in their pathogenesis.

In their review Iurca et al. present the involvement of tumor-associated macrophages (TAM) in the hallmarks of metastasis and their miRNA-related regulation with a focus on lung cancer Iurca et al. TAM have role in immunosuppression, angiogenesis and lymphangiogenesis, vessel intravasation and extravasation of cancer cells, and premetastatic niche formation. An indirect therapeutic approach on TAM can be also represented by regulation of miRNAs involved in their polarization and implicit oncogenic features such as studied miR-21 and miR-155, but also other miRNA less present in the current literature: miR-1207-5p, miR-193b, miR-320a, and others.

Kwon et al. group’s review is focused on interactions between cancer cells and stromal cells specifically macrophages *via* exosome containing miRNA. Exosomes are lipid bilayer membrane vesicles derived from the luminal membrane of multivesicular bodies, which are constitutively released by fusion with the cell membrane. Exosomes protect miRNA from degradation, enabling them to be stably expressed in the extracellular space. In this review the role of several exosomal miRNAs from tumor cells in the polarization of macrophages are discussed and the targets of these miRNAs are presented. Tumor-derived exosomes miRNA play role in inducing the M1- or M2-like polarization of macrophages in the tumor microenvironment. For example, exosomal miRNA-146a from hepatic cancer cells and exosomal miRNA-203 from colorectal cancer cells have oncogenic activity and induce the pro-tumoral M2 polarization of macrophages and accelerated cancer progress (2, 3). On the other hand, exosomal miRNA-19a-3p by inducing M1-macrophages polarization, suppresses breast cancer progression (4). Conversely, the effects of exosomal miRNAs from TAMs on cancer cell invasion, growth, and anti-cancer drug resistance are presented. M2 phenotype TAMs-derived exosome miRNAs could affect tumor growth, invasion/metastasis, and anti-cancer drug resistance. A high number of TAMs has been associated with the poor prognosis of cancers. Exosomes from cancer cells carrying miRNAs proven to confer anti-cancer drug-resistance could be targeted. Conversion of immunosuppressive TAMs into M1 macrophages can be used in combination with current anti-cancer immunotherapies.

Recent studies reported important role for the adaptator kinase Trib1 (Tribbles pseudokinase 1) in M2-like macrophage function, and its overexpression in prostate cancer. The original research article of Niespolo et al. is among the first to explore the Trib1 3’ UTR capacity to bind several miRNAs. Among them, miRNA-101-3p and miRNA-132-3p were found to regulate directly Trib1 expression and function, driving an inflammatory M1 phenotype in human macrophages. They also show that multiple miRNAs predicted to regulate Trib1 in prostate cancer show decreased expression leading to the hypothesis that this miRNA regulation leads to elevated Trib1 expression in this tumor.

Following pathogen endocytosis, macrophages/dendritic cells instruct the adaptive immune response, in parallel to antigen-presentation, by providing cytokine production to promote an inflammatory response when macrophages are polarized in the classical M1 pathway or an anti-inflammatory response when they are polarized in the alternative M2 pathway. Polarization decision relies on two main metabolic pathways controlled by miRNAs as reviewed by Nelson and O’Connell. Indeed, the M1 pro-inflammatory macrophage profile triggered *in vitro* by lipopolysaccharide (LPS) and interferon (IFN)-γ drives an aerobic glycolysis signal, known as Warburg Effect, that helps to maintain a cellular redox state in the cells with the help of two master transcription factors: nuclear factor kappa B (NF-κB) and hypoxia inducible factor 1α (HIF1α). This leads to the production of interleukin (IL)1-β, tumor necrosis factor (TNF)-α, IL-6, IL-12, IL-23, reactive oxygen species (ROS), L-arginine conversion into citrulline, and nitric oxide (NO) production. At the opposite, IL-4 and IL-13 polarized M2 anti-inflammatory macrophage cells use oxidative phosphorylation through a janus kinase (Jak)-3/signal transducer and activator of transcription (STAT)-6 dependent axis to drive wound healing, humoral immunity, angiogenesis, tissue remodeling, and to produce the immunosuppressive IL-10, IL-13 and transforming growth factor (TGF)-β cytokines. Additional pathways are important in M2 polarized macrophages such as AMP-activated protein kinase (AMPK) that promote fatty acid oxidation (FA0).

As a consequence, macrophage metabolic functions, polarization and cytokine production have to be tightly regulated, which can be achieved by miRNAs acting intracellularly or transport to distant cells *via* exosomes. At the metabolic level, shift from an oxidative phosphorylation to an aerobic signal is restrained by miRNA-146a that impedes mechanistic target of rapamycin (mTOR) signaling by interacting with TNF receptor-associated factor (Traf)-6 and miRNA-21 that controls phosphofructokinase (PFK)-M, an enzyme of the glycolytic pathway (5, 6). At the opposite, miRNA-125b by targeting the pro-apoptotic B-cell lymphoma-2 interacting killer (Bik) molecule promotes mitotic oxygen consumption, miRNA-33 dampens AMPK and in turn FAO, which both switch the metabolic programming into an aerobic glycolysis to support a pro-inflammatory activation. Downstream, M1 polarization can be repressed *via* (i) the control of the toll-like receptor (TLR) pathway (miRNA-146a controls Traf6 and interleukin-1 receptor-associated kinase (IRAK)-1; and Let-7e controls TLR-4 and IRAK-1); (ii) the control of the transcription factors NF-κB (miRNA-210) and HIF1-α (miRNA-17/20a); (iii) the control of cytokine production (Let-7b controls IL-12, IL-23 and TNF-α); and (iv) the control of inflammatory/interferon genes activated in an autocrine loop (miRNA-21 controls phosphatase and tensin homolog (PTEN) expression leading to serine/threonine-protein kinases (AKT) and STAT-3 activation, and miR-125b controls the interferon regulatory factor [IRF]-4). Such effect is counterbalanced by pro-M1 miRNAs that can act (i) on TLR signaling inhibitors through a downregulation of the protein tyrosine phosphatase activity (miRNA-155 controls suppressor of cytokine signaling [SOCS] and src homology 2 domain-containing protein tyrosine phosphatase [SHP]-I); (ii) on NF-κB/HIF1α activity through the respective control of their inhibitor: A20 for NF-κB (miRNA-125a and Let-7f) and factor-inhibiting HIF-1 **(**FIH)-1 for HIF1α (miRNA-125a); (iii) on silencing anti-inflammatory targets to induce IL-12 (miRNA-155) and IL-6 [Let-7abf through ten-eleven translocation (TET)-2 control)]; and (iv) on wound healing control [Let-7c through targeting CCAAT-enhancer-binding protein (C/EBP)-δ and p21-activated kinase (PAK)-1)]. Lastly, amplification/inhibition loops exist that are regulated by NF-κB (amplification loop with miRNA-155 followed by an inhibition loop with miRNA-146a and miRNA-21), HIF1α (amplification loop: miRNA-210 and miRNA-30c).

Expressed in both myeloid and lymphoid cells, miRNA-155 is the prototype of proinflammatory miRNA that contributes to drive M1 polarization, and to inhibit M2 polarization through IL-4/IL-13 pathway inhibition. Loss of miRNA-155 dampens the macrophage antiviral immune response, while its overexpression was associated with different inflammatory diseases as reviewed by Pasca et al. in this Research Topic. This includes autoimmune diseases (multiple sclerosis, rheumatoid arthritis, inflammatory bowel diseases), asthma, atherosclerosis, sarcoidosis and septic shock (7, 8). Interestingly, testing miRNA-155 represents a useful biomarker for inflammatory diseases as done in 26 clinical trials retrieved from ClinicalTrials.gov. The next step is to develop therapies based on miRNA-155 inhibition in inflammatory diseases that can be expected as small interfering RNA (siRNA) have received federal drug administration (FDA) approval in 2018 or by using locked nucleic acid-modified oligonucleotide inhibitors such as cobomarsen (MRG-106) (as reviewed in Kwon et al.).

MiRNA are also important modulators of cellular pathways with role in normal hematopoietic differentiation and miRNA expression is significantly correlated with the prognosis of hematopoietic malignancies, including AML. Oncogenic miRNAs correlate with poor prognosis, while tumor suppressor miRNAs, which inhibit the expression of proto-oncogenes, are correlated with a favorable prognosis (Neaga et al.). miRNAs are proposed as biomarkers for diagnosis and prognosis and are regarded as therapeutic approaches in many cancers, including AML.

In conclusion, miRNAs with epigenetic or modulatory activity, as well as with synergistic activity with chemotherapeutic agents, proved to be promising therapeutic targets in experimental, pre-clinical approaches. The clinical availability of emerging compounds with mimicking or suppressor activity provides the opportunity for future therapeutic targeting of miRNAs.

## Author Contributions

All authors listed have made a substantial, direct and intellectual contribution to the work, and approved it for publication.

## Conflict of Interest

The authors declare that the research was conducted in the absence of any commercial or financial relationships that could be construed as a potential conflict of interest.

## Publisher’s Note

All claims expressed in this article are solely those of the authors and do not necessarily represent those of their affiliated organizations, or those of the publisher, the editors and the reviewers. Any product that may be evaluated in this article, or claim that may be made by its manufacturer, is not guaranteed or endorsed by the publisher.
